# Phylogenetic Distribution of the Capsid Assembly Protein Gene (*g20*) of Cyanophages in Paddy Floodwaters in Northeast China

**DOI:** 10.1371/journal.pone.0088634

**Published:** 2014-02-12

**Authors:** Ruiyong Jing, Junjie Liu, Zhenhua Yu, Xiaobing Liu, Guanghua Wang

**Affiliations:** 1 Key Laboratory of Mollisols Agroecology, Northeast Institute of Geography and Agroecology, Chinese Academy of Science, Harbin, China; 2 Graduate University of Chinese Academy of Science, Beijing, China; 3 Heilongjiang BaYi Agricultural University, College of Life and Sci-technology, Daqing, China; J. Craig Venter Institute, United States of America

## Abstract

Numerous studies have revealed the high diversity of cyanophages in marine and freshwater environments, but little is currently known about the diversity of cyanophages in paddy fields, particularly in Northeast (NE) China. To elucidate the genetic diversity of cyanophages in paddy floodwaters in NE China, viral capsid assembly protein gene (*g20*) sequences from five floodwater samples were amplified with the primers CPS1 and CPS8. Denaturing gradient gel electrophoresis (DGGE) was applied to distinguish different *g20* clones. In total, 54 clones differing in *g20* nucleotide sequences were obtained in this study. Phylogenetic analysis showed that the distribution of *g20* sequences in this study was different from that in Japanese paddy fields, and all the sequences were grouped into Clusters α, β, γ and ε. Within Clusters α and β, three new small clusters (PFW-VII∼-IX) were identified. UniFrac analysis of *g20* clone assemblages demonstrated that the community compositions of cyanophage varied among marine, lake and paddy field environments. In paddy floodwater, community compositions of cyanophage were also different between NE China and Japan.

## Introduction

Viruses are recognized as the most abundant biological entities on earth [1], [2]. As mortality agents affecting heterotrophic and photosynthetic microbes, viruses play important roles in regulating the microbial population and community structure [3], mediating gene transfer between microorganisms [4], [5], and driving the global biogeochemical nutrient cycle [4], [5]. Viruses are supposed to be the greatest genomic reservoirs due to their great abundance and diversity [6], [7], [8]. Bacteriophages (phages) represent the majority of viruses in the natural environments [1], [2].

Cyanophages are viruses that are able to infect cyanobacteria. Unicellular cyanobacteria of the genera *Synechococcus* and *Prochlorococcus* are the most abundant forms of marine picoplankton [9], [10], whereas filamentous cyanobacteria such as *Nostoc*, *Anabaena*, *Cylindrospermum,* and *Phormidium* are dominant forms in freshwater [11]. Although several cyanophages that infect filamentous cyanobacteria have been isolated from freshwater with solid or liquid medium, research on their genetic diversity is limited [12], [13]. Currently, the knowledge of the genetic diversity of cyanophages is mainly based on the phages infecting oceanic *Synechococcus* and *Prochlorococcus* [14], [15], [16]. Most cyanophages are classified into the three-tailed phage families *Myoviridae*, *Podoviridae*, and *Siphoviridae*, among which cyanomyoviruses represent more than 80% of cyanophage isolated from the marine environments [6], [14].

Studying the diversity of phages has proven difficult because no universal genetic marker, analogous to the 16S or 18S rRNA gene used in microbial communities exists across all phage families [17]. However, recent results of phage genomics elucidated that some family-specific genes have been proposed for the evaluation of phage diversity [18]. Among these genes, *g20*, which encodes the capsid assembly protein of cyanomyoviruses, is commonly used as a biomarker to analyze the genetic diversity of the cyanophage community [14], [16], [19], [20]. Using the primers CPS1/CPS8, highly diverse *g20* fragments were discovered in marine and freshwater environments [14], [19], [20]. For example, cloning and sequencing analyses of six natural virus concentrations from estuarine and oligotrophic offshore environments revealed nine phylogenetic groups [19]. The use of this primer set and its modification (CPS4/G20-2 [21]; CPS1.1/CPS8.1 [22]) resulted in further grouping of cyanophage *g20* genes in various seawaters [20], [21], [22] and freshwaters [21], [23], [24].

Cyanobacteria are one of major microbial components in paddy fields and play an important role in maintaining soil fertility by fixing atmospheric N_2_ to ammonia [25]. A previous study indicated that cyanophage diversity in Japanese paddy floodwaters as estimated by the *g20* sequences distributed very broadly in a phylogenetic tree. The study also showed that the majority of the *g20* clones belonged to several unique paddy floodwater (PFW) groups, which were more closely related with the *g20* sequences from the freshwater environment than those from the marine environment [26]. Given that the distribution and assemblages of *g23*, another biomarker gene for assessing T4-type phages in paddy fields were different between Japan and Northeast (NE) China [27], we speculated that cyanophage communities in paddy fields might also be different between the two countries and unrevealed cyanophage *g20* might exist in paddy fields in NE China. In this study, we surveyed the *g20* sequences in five paddy floodwater samples obtained from NE China. The aims of this study were to (1) evaluate the phylogenetic position of obtained *g20* sequences relative to previously reported sequences, and (2) compare the *g20* assemblages in paddy floodwaters of NE China with those in Japanese paddy fields, and freshwater and marine water environments.

## Materials and Methods

### Paddy floodwater sampling

Paddy floodwater samples were collected from five paddy fields in NE China from July 14 to 21 in 2011. The five paddy fields included Da-An (DA) (45°36' N, 123°50' E) in Jilin province, and Sui-Hua (SH) (46°43' N, 126°59' E), Jian-San-Jiang (JSJ) (47°14' N, 132°33' E), A-Cheng (AC) (45°28' N, 126°58' E) and Lin-Dian (LD) (47°18' N, 124°37' E) in Heilongjiang province. At each sampling location, we had obtained the landowners’ permission prior to conducting the study, and sampling procedures did not impact endangered or protected species in environments. Rice seedlings were transplanted from May 20 to June 10 in 2011 and were managed with conventional practices. Approximately 500 mL of floodwater was collected from several sites in the middle part of each field site. Water samples were kept in a container with an ice bag and transported to the laboratory within 12 h.

When these water samples arrived at the laboratory, they were centrifuged immediately at 8, 000 × *g* for 30 min at 4°C to remove soil particles, plankton, and bacteria. The samples were then filtrated through a 0.4-µm and 0.2-µm cellulose filter to completely remove bacteria. Virus-size particles were collected on 0.03-µm filter membrane (Nuclepore Track-Etch Membrane, Whatman, UK) using vacuum filtration. The filter was crushed carefully with forceps and put into a 2-mL sterilized tube with 700 µL 10 mM Tris-HCl buffer (pH 7.5).

### DNA extraction and PCR amplification

The crushed filter in the tube was treated with DNase and RNase (40 µg mL^−1^ each) for 5 h at 37°C to decompose free DNA and RNA. Then, 38 µL 10% SDS, 7.5 µL 1M Tris-HCl, 15 µL 0.5 M EDTA, and 2 µL proteinase K (10 mg mL^−1^) were added to the tube, which was vortexed for 2 min and incubated for 30 min at 55°C with gentle shaking by hand every 10 min. At the end of incubation, 140 µL 5 M NaCl and 150 µL CTAB/NaCl solutions were added into the tube, which was further incubated for 10 min at 65°C [28]. Viral DNA was extracted twice with PCI solution (phenol:chloroform:isoamyl alcohol =  25:24:1, v/v) and once with CIA solution (chloroform:isoamyl alcohol = 24:1, *v/v*). The aqueous phase was treated with 0.6 volume of cold isopropanol (-20°C) and centrifuged at 15,000 × *g* for 20 min at 4°C to obtain a DNA pellet. The precipitated DNA was washed with 70% ethanol, dried, and resuspended in TE buffer (10 mM Tris-HCl, 1 mM EDTA, pH 8.0).

The capsid assembly protein gene *g20* was amplified with the primers CPS1 (5^'^-GTA GWA TTT TCT ACA TTG AYG TTG G-3') and CPS8 (5'-AAA TAY TTD CCA ACA WAT GGA-3') [19]. Briefly, 50 µL PCR mixture contained 0.5 µL forward and reverse primers (50 pmol each), 1-2 µL DNA template, 5 µL dNTPs (2.5 mM each; TaKaRa, Dalian, China), 5 µL rTaq buffer (TaKaRa, Dalian, China), 0.5 µL rTaq polymerase (5U µL^−1^, TaKaRa, Dalian, China) and was filled to the required volume with sterile MilliQ water (36.5-37.5 µL). The negative control contained all reagents and sterile MilliQ water without the template. PCR amplification was performed by a thermal cycle PCR machine (ABI 9700, Foster City, CA, USA) at 94°C for 5 min (initial denaturation), followed by 35 cycles of 94°C for 45 sec, 35°C for 45 sec, and 72°C for 1 min, with a final extension at 72°C for 5 min.

### Cloning, denaturing gradient gel electrophoresis (DGGE) and sequencing

A PCR product of approximately 600 bp in length was cut from a 2% agarose gel and purified using the QIAquick Gel Extraction Kit (Qiagen, Crawley, UK). The purified DNA was cloned into the pMD18-T plasmid vector (TaKaRa, Dalian, China) and transformed into competent cells of *Escherichia coli* DH5α according to the manufacturer’s instruction. Approximately 50 clones from each transformation were chosen from white clones and amplified with primers CPS1 and CPS8. The PCR program was the same as described above, except for reducing the cycle number to 28. Six microliters of the PCR product of a positive clone was used for DGGE according to the previously described method [26]. Bands with the same mobility on a DGGE gel were considered as the same clones. Plasmid DNA from different clones was harvested from an overnight culture of *E. coli* DH5α and submitted to a commercial company (BGI, Shenzhen, China) for sequencing.

### Phylogenetic analysis

Clone nucleotide sequences were translated to deduced amino acid sequences using the EMBOSS Transeq program on the European Bioinformatic Institute website (http://www.ebi.ac.uk/). The closest relatives of *g20* clones were examined using the BLAST search program on the NCBI website (http://www.ncbi.nlm.nih.gov/) at the amino acid level. The identities of amino acid sequences among these clones obtained in this study were analyzed using the ClustalW program available on the DNA Data Bank of Japan (DDBJ) website (http://www.ddbj.nig.ac.jp/). The phylogenetic position of *g20* clones obtained in this study was firstly compared with that of *g20* sequences obtained from Japanese paddy floodwaters and soils [26], [29]. Their positions were further compared with that of the closest relatives of representative *g20* fragments retrieved from GenBank, and of three outgroup non-cyanophage *g20* sequences from Coliphage T4(AF158101), Vibriophage KVP40 (AB020525), and *Aeromonas* phage Aeh1 (AY266303) [16]. The amino acid sequences were aligned with ClustalX 1.81 [30]. A neighbor-joining tree was constructed using MOLECULAR EVOLUTIONARY GENETIC ANALYSIS software (MEGA 4.0; [31]) with 1,000 bootstrap replicates.

To evaluate whether the distributions of *g20* clone assemblages were related to their obtained environments, two unweighted UniFrac statistical analyses were performed in this study using software available at http://bmf.colorado.edu/unifrac/ [32]. The first analysis was conducted using the *g20* sequences obtained in this study and those obtained from Japanese paddy fields to test whether *g20* assemblages vary between the two countries in similar environments. The second analysis involved *g20* sequences from paddy fields, lake freshwaters, and marine waters to detect whether *g20* assemblages vary between different environments. Sampling sites and the number of *g20* sequences used in those two unweighted UniFrac analyses are shown in Table S1.

The DNA sequences of *g20* obtained in this study have been deposited in the NCBI database with accession numbers from KF017951 to KF018004.

## Results

### Closest relatives of *g20* genes

We obtained 154 positive clones by PCR amplification with the primers CPS1 and CPS8. After analyzing all of the clone positions of an individual sample in DGGE gel and deleting clones with identical nucleotide sequences, 54 clones with different *g20* sequences were obtained in this study. Among these clones, 11, 10, 12, 10, and 10 clones were obtained from the locations JSJ, AC, DA, LD, and SH, respectively. The length of *g20* fragments (excluding primer parts) varied among clones: 18 clones were 552-bp long (33%), and 36 clones were 546-bp long (67%).

A BLAST search for the closest relatives at the amino acid level revealed that seven clones had the highest identities (from 75% to 94%) to the *g20* clones from Japanese paddy floodwater; four clones had the highest identities (from 81% to 92%) to clones from Japanese paddy soils; three clones had the highest identities (from 73% to 74%) to clones from oceanic waters; six clones had the highest identities (from 67% to 87%) to clones from lake freshwaters; and 34 clones had the highest identities (from 67% to 88%) to clones from the Kranji reservoir in Singapore (Table 1). The identity among the clones in this study ranged from 51% (PFW-JSJ-9 and PFW-JSJ-11; PFW-JSJ-11 and PFW-LD-8) to 100% (PFW-AC-2 and PFW-LD-3; PFW-JSJ-1 and PFW-JSJ-4; PFW-SH-2 and PFW-DA-1).

**Table 1:** Closest relatives of sequenced *g20* clones from different paddy floodwaters at the amino acid level.

Clone name	Length Amino acid	Closest relative	Accession No.	Identity %	Alignment	Groups	Sources	References
PFW-AC-1	183	PFW-NoF21	BAG85069	83	152/184	PFW-IX	Paddy floodwater	Wang et al., 2010
PFW-AC-2	183	KRC0908M3	AGL61483	83	151/183	PFW-IX	Kranji Reservoir	Yeo and Gin, unpublished 2013
PFW-AC-3	183	KRA0908M3	AGL61424	83	152/183	PFW-IX	Kranji Reservoir	Yeo and Gin, unpublished 2013
PFW-AC-4	183	KRC0908M3	AGL61483	83	151/183	PFW-IX	Kranji Reservoir	Yeo and Gin, unpublished 2013
PFW-AC-5	183	KRC0908M3	AGL61483	83	151/183	PFW-IX	Kranji Reservoir	Yeo and Gin, unpublished 2013
PFW-AC-6	183	KRC0908M3	AGL61483	83	151/183	PFW-IX	Kranji Reservoir	Yeo and Gin, unpublished 2013
PFW-AC-7	183	KRC0908M3	AGL61483	82	151/183	PFW-IX	Kranji Reservoir	Yeo and Gin, unpublished 2013
PFW-AC-8	183	KRA0908M3	AGL61424	83	151/183	PFW-IX	Kranji Reservoir	Yeo and Gin, unpublished 2013
PFW-AC-9	183	PFW-NoF21	BAG85069	83	152/184	PFW-IX	Paddy floodwater	Wang et al., 2010
PFW-AC-10	183	KRC0908M3	AGL61483	82	150/183	PFW-IX	Kranji Reservoir	Yeo and Gin, unpublished 2013
PFW-JSJ-1	181	BES02B-28	AAW48772	74	133/180	ungrouped	Arctic Ocean	Short and Suttle, 2005
PFW-JSJ-2	183	KRC0908M3	AGL61483	82	150/183	PFW-IX	Kranji Reservoir	Yeo and Gin, unpublished 2013
PFW-JSJ-3	181	PFW-CM12	BAG85104	94	171/181	PFW-VI	Paddy floodwater	Wang et al., 2010
PFW-JSJ-4	181	BES02B-28	AAW48772	74	133/180	ungrouped	Arctic Ocean	Short and Suttle, 2005
PFW-JSJ-5	181	KRC0209M1	AGL61498	81	147/181	ungrouped	Kranji Reservoir	Yeo and Gin, unpublished 2013
PFW-JSJ-6	181	KRA1008M5	AGL61431	78	142/181	ungrouped	Kranji Reservoir	Yeo and Gin, unpublished 2013
PFW-JSJ-7	181	BES02B-28	AAW48772	73	132/180	ungrouped	Arctic Ocean	Short and Suttle, 2005
PFW-JSJ-8	183	KRC0908M3	AGL61483	82	150/183	PFW-IX	Kranji Reservoir	Yeo and Gin, unpublished 2013
PFW-JSJ-9	181	VC64_E2	ABC49841	67	122/182	ungrouped	Lake Erie	Wilhelm et al., 2006
PFW-JSJ-10	181	LAB_g20_b12_E4	AGN88770	87	158/181	ungrouped	Freshwater	Zhong and Jacquet, 2013
PFW-JSJ-11	181	AnCf-Apr11-5	BAJ07513	92	166/181	Group FPS-I	Paddy field soil	Wang et al., 2011
PFW-LD-1	181	PFW-CM12	BAG85104	94	171/181	PFW-VI	Paddy floodwater	Wang et al., 2010
PFW-LD-2	183	KRC0908M3	AGL61483	82	150/183	PFW-IX	Kranji Reservoir	Yeo and Gin, unpublished 2013
PFW-LD-3	183	KRC0908M3	AGL61483	83	151/183	PFW-IX	Kranji Reservoir	Yeo and Gin, unpublished 2013
PFW-LD-4	183	KRC0908M3	AGL61483	82	150/183	PFW-IX	Kranji Reservoir	Yeo and Gin, unpublished 2013
PFW-LD-5	183	KRC0908M3	AGL61483	83	151/183	PFW-IX	Kranji Reservoir	Yeo and Gin, unpublished 2013
PFW-LD-6	183	KRC0908M3	AGL61483	82	150/183	PFW-IX	Kranji Reservoir	Yeo and Gin, unpublished 2013
PFW-LD-7	181	LAB_g20_b24_A12	AGN88792	88	159/181	ungrouped	Freshwater	Zhong and Jacquet, 2013
PFW-LD-8	181	KRA0209M4	AGL61448	79	143/180	ungrouped	Kranji Reservoir	Yeo and Gin, unpublished 2013
PFW-LD-9	183	KRC0908M3	AGL61483	82	150/183	PFW-IX	Kranji Reservoir	Yeo and Gin, unpublished 2013
PFW-LD-10	181	LAB_g20_b24_A12	AGN88792	88	158/181	PFW-IV	Freshwater	Zhong and Jacquet, 2013
PFW-DA-1	181	KRA1008M5	AGL61431	83	148/179	PFW-VIII	Kranji Reservoir	Yeo and Gin, unpublished 2013
PFW-DA-2	181	KRC1008M3	AGL61488	67	1122/181	PFW-VII	Kranji Reservoir	Yeo and Gin, unpublished 2013
PFW-DA-3	181	KuCf-Apr13-7	BAJ07470	84	149/178	PFW-VIII	Paddy field soil	Wang et al., 2011
PFW-DA-4	181	KRA1108M3	AGL61434	87	158/181	PFW-I	Kranji Reservoir	Yeo and Gin, unpublished 2013
PFW-DA-5	181	KRA1108M3	AGL61434	87	158/181	PFW-I	Kranji Reservoir	Yeo and Gin, unpublished 2013
PFW-DA-6	181	PFW-CF1	BAG85081	91	165/181	PFW-II	Paddy floodwater	Wang et al., 2010
PFW-DA-7	181	PFW-CM29	BAG85121	83	149/180	PFW-IV	Paddy floodwater	Wang et al., 2010
PFW-DA-8	181	KRB1208M1	AGL61464	80	142/178	ungrouped	Kranji Reservoir	Yeo and Gin, unpublished 2013
PFW-DA-9	181	KRC1008M3	AGL61488	68	123/181	PFW-VII	Kranji Reservoir	Yeo and Gin, unpublished 2013
PFW-DA-10	181	PFW-CM29	BAG85121	75	135/181	PFW-IV	Paddy floodwater	Wang et al., 2010
PFW-DA-11	181	KRC1008M3	AGL61488	67	122/181	PFW-VII	Kranji Reservoir	Yeo and Gin, unpublished 2013
PFW-DA-12	181	KRA1108M3	AGL61434	87	158/181	PFW-I	Kranji Reservoir	Yeo and Gin, unpublished 2013
PFW-SH-1	181	SPM02-24	AAW48769	71	126/177	ungrouped	Shore Pond Mat	Short and Suttle, 2005
PFW-SH-2	181	KRA1008M5	AGL61431	83	148/179	PFW-VIII	Kranji Reservoir	Yeo and Gin, unpublished 2013
PFW-SH-3	181	KuCf-Apr13-7	BAJ07470	84	148/177	PFW-VIII	Paddy field soil	Wang et al., 2011
PFW-SH-4	181	KRA1008M5	AGL61431	82	147/179	PFW-VIII	Kranji Reservoir	Yeo and Gin, unpublished 2013
PFW-SH-5	181	KRA1008M5	AGL61431	82	147/179	PFW-VIII	Kranji Reservoir	Yeo and Gin, unpublished 2013
PFW-SH-6	181	SPM02-24	AAW48769	71	125/177	ungrouped	Shore Pond Mat	Short and Suttle, 2005
PFW-SH-7	181	KRA1008M5	AGL61431	82	147/179	PFW-VIII	Kranji Reservoir	Yeo and Gin, unpublished 2013
PFW-SH-8	181	KuCf-Jul26-5	BAJ07489	81	143/177	PFW-VIII	Paddy field soil	Wang et al., 2011
PFW-SH-9	181	KRA1008M5	AGL61431	82	146/179	PFW-VIII	Kranji Reservoir	Yeo and Gin, unpublished 2013
PFW-SH-10	181	KRA1008M5	AGL61431	82	147/179	PFW-VIII	Kranji Reservoir	Yeo and Gin, unpublished 2013
PFW-SH-11	181	KRA1008M5	AGL61431	82	147/179	PFW-VIII	Kranji Reservoir	Yeo and Gin, unpublished 2013

### Phylogeny of *g20* genes

**Figure 1:**
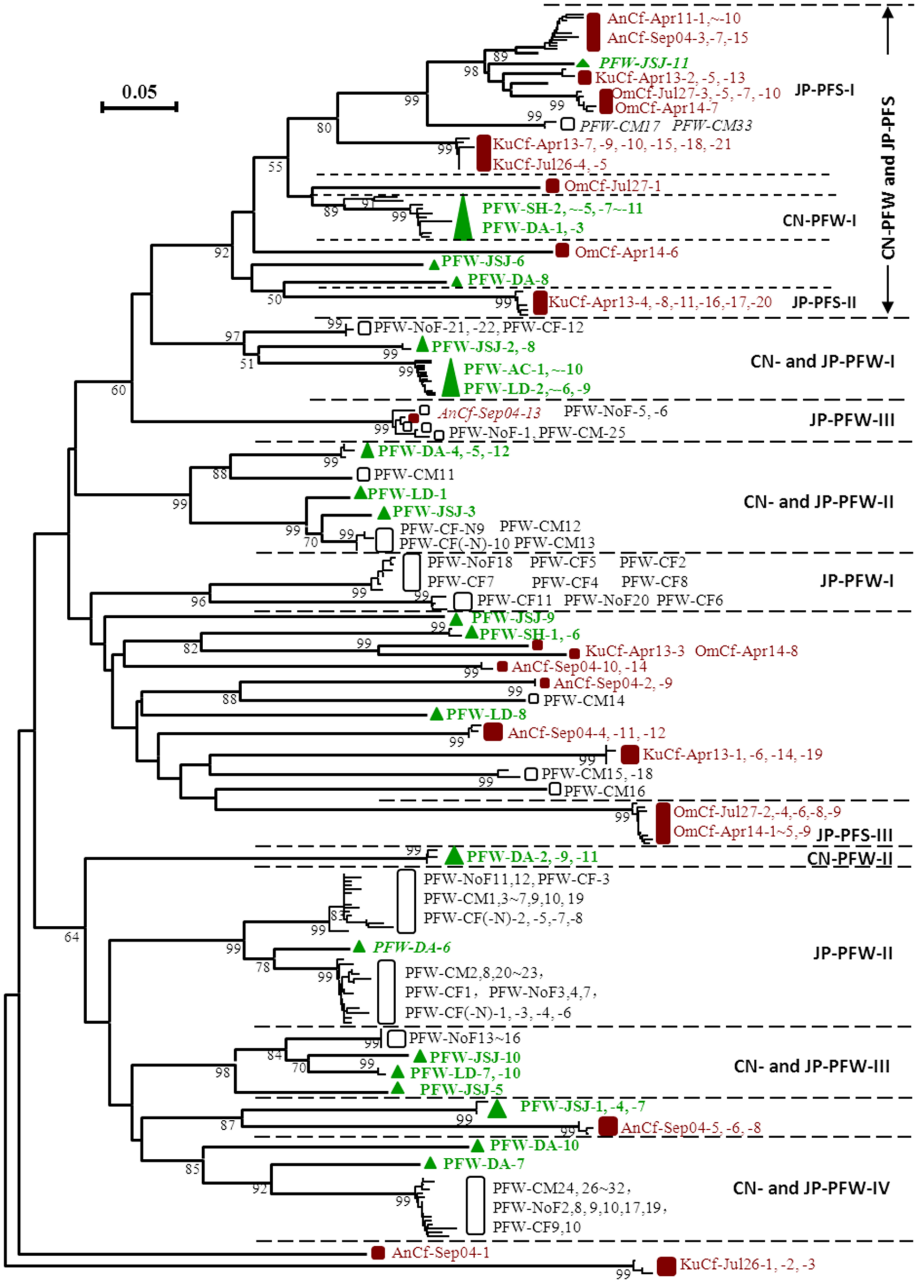
Neighbor-joining phylogenetic tree showing the relationship of *g20* amino acid sequences from paddy floodwaters in NE China with those from Japanese paddy floodwaters (Wang et al. 2010) and paddy field soils (Wang et al.2011). *Brown* and *white square boxes* indicate *g20* clones obtained from paddy field soils in Japan and paddy floodwaters in Japan, respectively; *green triangles* indicate *g20* clones obtained from paddy floodwaters in NE China; *JP* and *CN* represent Japan and China, respectively; *PFW* and *PFS* represent paddy floodwater and paddy field soil, respectively. Bootstrap values <50 are not shown. The scale bar represents the number of amino acid substitutions per residue.

The phylogenetic relationships of the *g20* clones obtained in this study with those observed from Japanese paddy fields, including paddy floodwaters and soils [26], [29], are shown in Fig. 1. Based on the overall architecture of the tree, at least 10 clusters with high bootstrap support values were formed. Among these clusters, two clusters (CN-PFW-I and CN-PFW-II) consisted of 11 and 3 clones, respectively, exclusively obtained in this study; four clusters (CN- and JP-PFW-I∼CN- and JP-PFW-IV) consisted of 18, 5, 4, and 2 clones obtained in this study and Japanese paddy floodwaters; three clusters (JP-PFW-I∼JP-PFW-III) mainly contained clones obtained from Japanese paddy floodwaters; three clusters (JP-PFS-I∼JP-PFS-III) contained clones obtained from Japanese paddy soils. Additionally, subclusters CN-PFW-I, JP-PFS-I and JP-PFS-II formed a larger cluster (CN-PFW and JP-PFS) with high bootstrap support (92%) at the top of the tree, which consisted of 14 clones obtained in this study, and clones mainly obtained from Japanese paddy soils. The few exceptional clones that fell outside of the above clusters were marked with italic letters in Fig. 1. In addition, in the middle of the tree, four *g20* clones observed in this study formed several weakly supported clades with *g20* clones from Japanese paddy floodwaters and soils.

**Figure 2:**
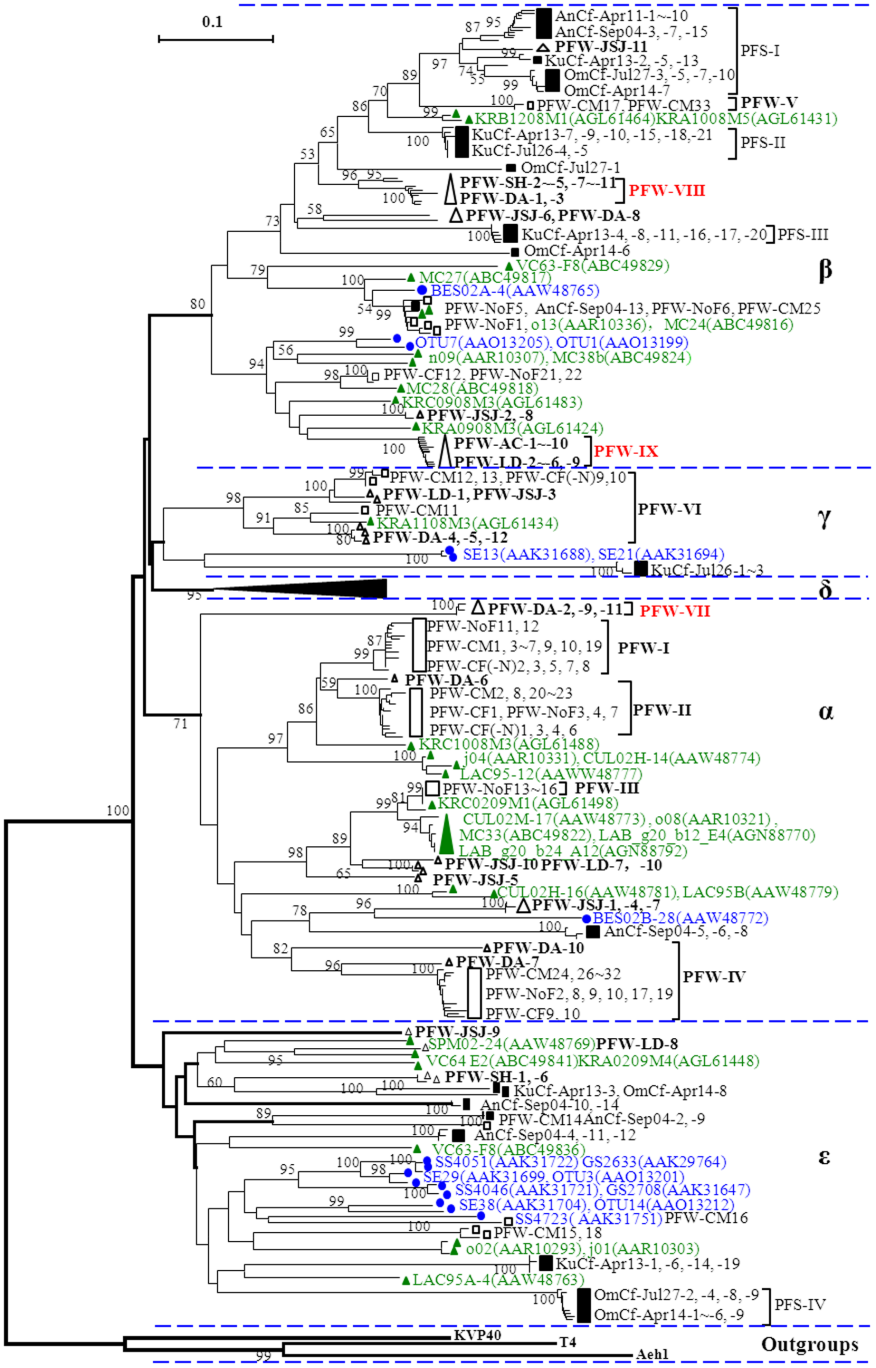
Neighbor-joining phylogenetic tree showing the relationships of *g20* amino acid sequence from paddy floodwaters in NE China with from those from lake freshwaters (Dorigo et al. 2004; Short and Suttle 2005; Wilhelm et al. 2006; Zhong and Jacquet 2013; Yeo and Gin, unpublished data which were submitted in Jan 15, 2013), paddy floodwaters in Japan (Wang et al. 2010), paddy field soils in Japan (Wang et al. 2011) and marine waters (Fuller et al. 1998; Zhong et al. 2002; Marston and Sallee 2003; Wang and Chen 2004; Mann et al. 2005; Short and Suttle 2005; Li and Li, unpublished data which were submitted in Jun 16, 2013). *Green triangles* and *blue circles* indicate *g20* clones obtained from lake freshwater and marine water, respectively; *Black* and *white square boxes* indicate *g20* clones obtained from paddy field soils in Japan and paddy floodwaters in Japan, respectively; *White triangles* indicate *g20* clones obtained from paddy floodwaters in NE China. The *number in parentheses* denotes the accession number of amino acid sequences in the NCBI website. Bootstrap values <50 are not shown. The scale bar represents the number of amino acid substitutions per residue.

Fig. 2 showed the phylogenetic relationships of the *g20* clones obtained in this study with the representative *g20* clones and isolated phages from lake freshwaters [21], [23], [24] and marine waters [14], [19], [20], [21], [23], [24], [33], [34], and all *g20* clones from Japanese paddy floodwaters and soils [26], [29]. The tree revealed that the *g20* clones obtained in this study were distributed into four major clusters (α, β, γ and ε). In this study, the grouping of *g20* clones followed that of the previous reports [26], [29].

Cluster α was a large and weakly supported (71%) cluster, and 13 clones obtained in this study fell into this cluster. Within this cluster, clones PFW-DA-2, PFW-DA-9 and PFW-DA-11 formed a small branch far from other clones. In addition, these three clones had the highest identity of 67% to 68% to clone KRC1008M3 obtained from Kranji reservoir in Singapore (Table 1). Therefore, this branch was designed as a new PFW group, named PFW-VII. Clone PFW-DA-6 had the highest identity of 91% to clone PFW-CF1 (Table 1) and fell into the PFW-II group. Clones PFW-DA-7 and PFW-DA-10 had the highest identities of 83% and 75%, respectively to clone PFW-CM29 (Table 1) and were clustered into the extended PFW-IV group. Other clones were clustered closely with *g20* clones from paddy fields and marine waters or lake freshwaters.

Cluster β was a large and strongly supported (80%) cluster, and 32 clones obtained in this study were grouped into this cluster. Within this cluster, two small branches consisted of 11 and 16 clones exclusively obtained in this study and were designed as new PFW groups of PFW-VIII and PFW-IX, respectively. The clones in the two new designed groups had the highest identity of 81∼84% and 82∼83% to clones obtained from the Kranji reservoir in Singapore, Japanese paddy floodwaters and paddy field soils, respectively (Table 1). Clone PFW-JSJ-11 had the highest identity of 92% to the clone AnCf-Apr11-5 in a Japanese paddy field soil (Table 1) and was clustered into PFS-I group. Clones PFW-JSJ-6 and PFW-DA-8 were clustered close to the PFS-III group, and clones PFW-JSJ-2 and PFW-JSJ-8 were clustered close to clones from Japanese paddy floodwaters or lake freshwaters.

Cluster γ was a weak bootstrap supported (13%) cluster contained two clones (SE13 and SE21) from the surface water of a Savannah estuary [19], three clones from paddy field soils in Japan, one clone from Kranji reservoir in Singapore, five clones from the current study and five clones from Japanese paddy floodwaters. Except for clones SE13, SE21 and three clones from paddy field soils in Japan, those 11 clones formed a strongly supported (98%) cluster that was previously designed as PFW-VI [26]. Clones PFW-DA-4, PFW-DA-5, PFW-DA-12, PFW-JSJ-3, and PFW-LD-1 had the highest identities (87% and 94%) to clone KRA1108M3 from the Kranji reservoir and PFW-CM12 from Japanese paddy floodwater, respectively (Table 1).

Cluster ε was also a weakly supported (17%) cluster that contained four clones obtained in this study and clones from lake waters, Japanese paddy floodwaters and soils, and marine waters [19], [20], [23], [24], [26], [29]. Within this cluster, clones PFW-SH-1 and PFW-SH-6 had the highest identity (71%) to clone SPM02-24 obtained from a shore pond cyanobacterial mat in the Arctic Ocean (Table 1). Clones PFW-JSJ-9 and PFW-LD-8 had the highest identities (67% and 79%) to clones VC64-E2 from Lake Erie, Canada and KRA0209M4 from the Kranji reservoir, Singapore, respectively (Table 1). No clone obtained in this study fell into Cluster δ, even though two subclusters (CSP-PFW1 and CSP-PFW2) obtained from Japanese paddy floodwaters belonged to Cluster δ [26].

### UniFrac analysis of *g20* assemblage

**Figure 3:**
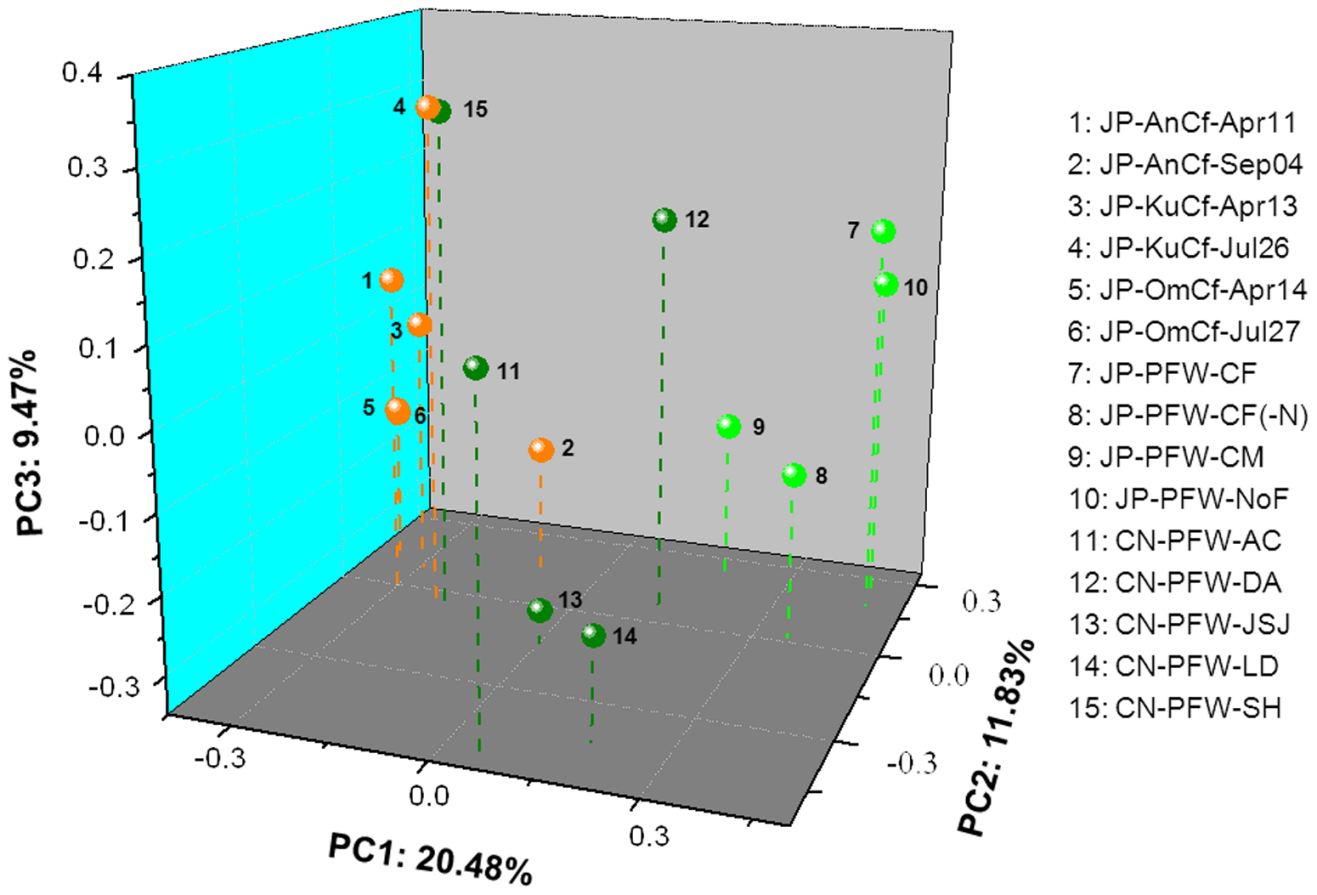
Three-dimensional principal coordinate analysis of *g20* clone sequences of cyanophage communities obtained from paddy floodwaters in NE China (*dark green circles*) and from Japanese paddy floodwaters (*light green circles*) and paddy soils (*brown circles*). The *percentages* in the axis labels represent the percentage of variation explained by the principal coordinates.

The *g20* assemblages in this study were compared with those from Japanese paddy floodwaters and soils using UniFrac analysis [32]. The three-dimensional plot of principal coordinate analysis (PCoA) based on PC1/PC2/PC3 showed that four out of five points of paddy floodwater samples from NE China were located separately from points of Japanese paddy floodwaters and soils, with the exception of PFW-SH overlapping with the Japanese soil of KuCf-Jul26 (Fig. 3). However, the *P*-value test demonstrated that the *g20* assemblages in paddy floodwater samples from NE China, including the sample of PFW-SH, were significantly different from those in samples from Japanese paddy floodwaters and soils (*P*<0.05) (Table S2).

**Figure 4:**
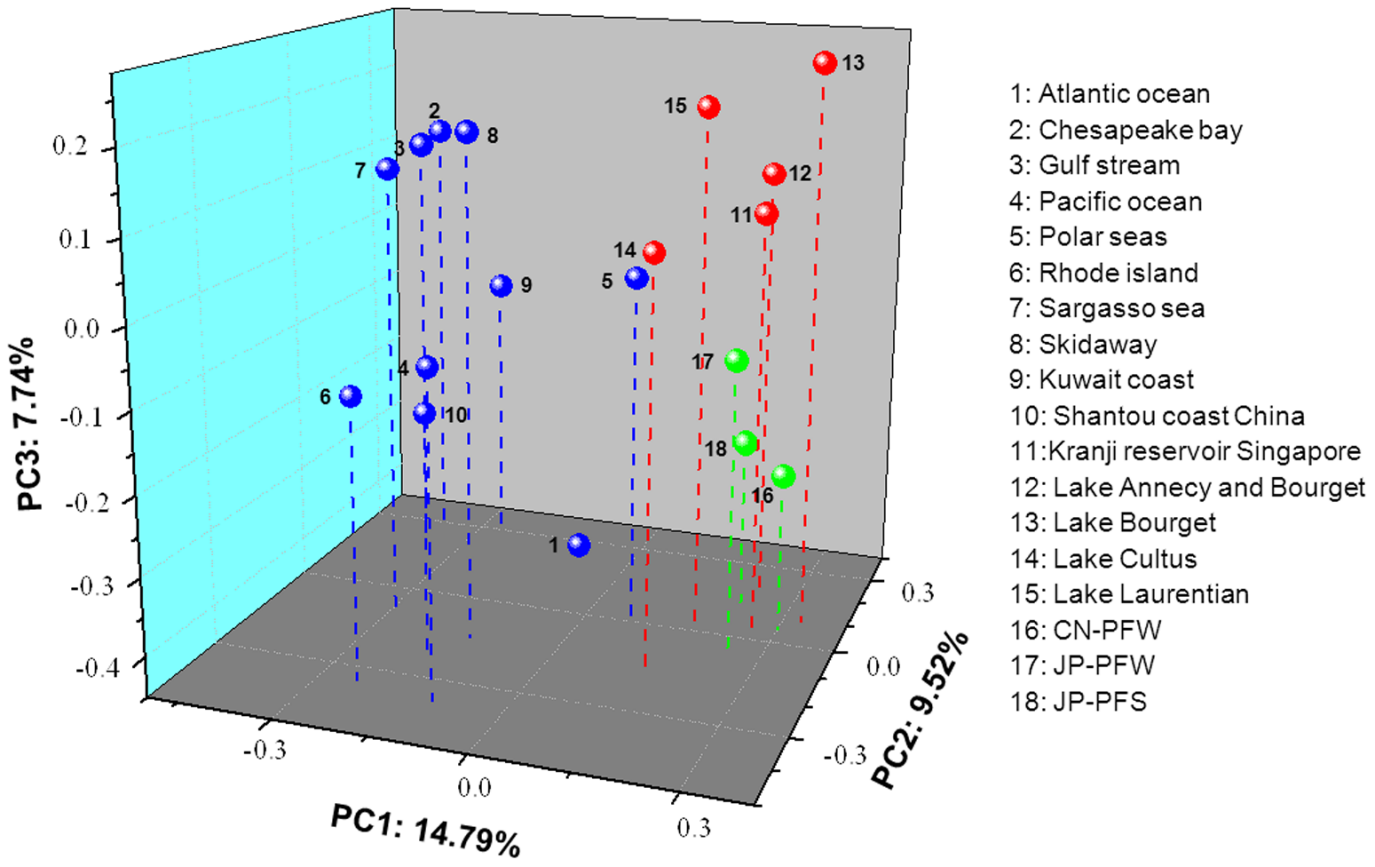
Three-dimensional principal coordinate analysis of *g20* clone sequences of cyanophage communities obtained from marine waters (*blue circles*), lake freshwaters (*red circles*), and paddy fields (*green circles*). The *percentages* in the axis labels represent the percentages of variation explained by the principal coordinates.

In order to compare the *g20* assemblages in paddy fields with those in other environments, all paddy floodwater samples from NE China were considered as one point (CN-PFW), and all paddy floodwater and soil samples from Japan were considered as two points (JP-PFW and JP-PFS). The *g20* clone assemblages of the three paddy field points were further compared with those from lake freshwaters and marine waters using UniFrac analysis (Fig. 4). The three-dimensional PCoA plot showed that three points of paddy fields (CN-PFW, JP-PFW, and JP-PFS) were located more closely to five points of freshwater lakes (Cultus, Bourget, and Laurentian, Kranji Reservoir in Singapore, and Lake Annecy and Bourget) than 10 points of oceans (Atlantic Ocean, Chesapeake Bay, Pacific Ocean, Polar Seas, Rhode island, Sargasso Sea, Skidaway, Kuwait coast, Shantou coast in China and Gulf Stream) around the world (Fig. 4). The *P*-value test indicated that the *g20* assemblages of paddy fields, including waters and soils, were significantly different from those of lake and marine environments (*P*<0.05) (Table S3).

## Discussion

### Phylogenetic position of *g20* genes in paddy floodwaters in NE China

Previous studies demonstrated that the distribution of *g23* gene of the T4-type phages was different among freshwaters, marine waters, paddy field soils, and upland black soils [35], [36]. Even in the similar environment of paddy field, the distribution of *g23* genes was also distinctly different between Japan and NE China, and several specific groups of T4-type phages were observed in the two countries [36]. Thus, we concluded that the T4-type phage communities in terrestrial environments are determined by both biogeographic and ecological processes [35]. However, we do not know whether this tendency is applicable to other phage families. In this study, we surveyed *g20* sequences in paddy floodwaters in NE China. Although the neighbor-joining tree showed that many of the clones obtained in this study fell into clusters containing clones previously observed in Japanese paddy floodwaters or paddy soils (Fig.1), there were three unique cluster/subclusters (PFW-VII∼PFW-IX) consisting of clones mainly from paddy floodwaters in NE China (Fig.2) (55.6% of paddy floodwater clones). In addition, there were also three clusters containing *g20* clones exclusively from Japanese paddy floodwaters and three clusters consist of clones exclusively from Japanese paddy soils (Fig.1). These findings suggested that similar to *g23* gene, the distribution of *g20* genes in paddy floodwater might also be different between Japan and NE China, even though the sample sizes of both studies were relatively small.

A previous study indicated that 77 different *g20* clones from Japanese paddy floodwater were distributed into five major clusters (α∼ε) with clones and isolated phages from freshwater and marine water, and the majority of clones formed eight unique paddy floodwater groups (PFW-I∼PFW-VI, CPS-PFW1, CPS-PFW2) within the major clusters [26]. Furthermore, 70 different *g20* clones from Japanese paddy field soils were distributed into Clusters α, β and ε, and four paddy field soil-specific subclusters (PFS-I∼IV) were formed within Clusters β and ε [29]. In this study, approximately 24%, 59%, 9%, and 7% of the obtained clones were distributed into the previously designated Clusters α, β, γ, and ε, respectively (Fig. 2). No clone fell into Cluster δ, also named as Cluster CSP, which was previously designated by Short and Suttle (2005). Cluster δ contained all of the *g20* sequences of cyanophages infecting *Synechococcus* and *Prochlorococcus*, *g20* clones collected from marine and freshwater environments, and 9 clones from Japanese paddy floodwaters [29]. Moreover, within Clusters α and β, three small clusters (PFW-VII∼PFW-IX) were designated in this study, but no clone from Japanese paddy floodwater or soil fell into these groups (Fig. 2). These findings further indicated that the cyanophage communities in paddy floodwater might be different between the two countries.

### Cyanophage host of *g20* genes in paddy floodwater

Although several *g20* specific clusters were obtained from paddy floodwater, we were still puzzled where these *g20* sequences in those clusters came from, because no representative *g20* sequences of a known phage fell into those environmental clusters. Short and Suttle (2005) doubted that environmental *g20* sequences outside of the CSP group were not from cyanophages [21]. However, there has been no direct evidence showing that *g20* sequences of non-cyanophages can be amplified with the primers CPS1/CPS8 till now. Therefore, we deduced that most PFW clones obtained with the primers CPS1/CPS8, if not all, could be regarded as cyanophage genes according to the work of Sullivan et al. [37]. The wide distributions of PFW clones suggested that various cyanobacteria, including *Synechococcus*, might be the hosts of phages in paddy floodwater, although most of these host cyanobacteria are still unknown.

Although cyanobacterial communities in paddy floodwaters were not investigated in this study, several studies found that their communities in paddy fields changed with location and time [38], [39], as well as with soil pH and pesticides [40]. Dozens of cyanophages infecting filamentous cyanobacteria have been isolated from freshwaters [13], [41], [42], [43], the information on their *g20* genes is still limited [12], [13]. Baker et al. (2006) found that the primers CPS1/CPS2 and CPS1/CPS8 failed in the amplification of *Anabaena* phages AN-15, A-1(L) and N-1 [12]. In contrast, Deng and Hayes (2008) successfully amplified *g20* gene of phage P-Z1 infecting *Planktothrix rubescens* BC9307 using CPS1/CPS2, but they did not test amplification with primers CPS1/CPS8 [13]. Because high genetic diversity of picocyanobacteria, including *Synechococcus*, has been detected in freshwater [44] and paddy floodwater (Wang et al., unpublished data), we are still unsure whether *g20* clones obtained from paddy floodwater originate from cyanophages infecting filamentous cyanobacteria. Further research using traditional culture-dependent methods should be considered to resolve this puzzle.

### Comparison of *g20* assemblages of cyanophage community in paddy floodwater with those in other environments

Cyanophage communities, as evaluated by *g20* assemblages, in Japanese paddy field were different between soil and paddy floodwater [29]. In this study, we further found that the points of *g20* assemblages in paddy floodwaters of NE China were not randomly distributed in the three-dimensional PCoA plot. Four of five points could be considered as a group, which was located more closely to Japanese paddy floodwaters than Japanese paddy soils (Fig.3). In addition, the *P*-test results clearly showed that the *g20* assemblages in paddy floodwaters of NE China were significantly different from those in both Japanese paddy floodwaters and soils (Table S2). This finding indicated that, although *g20* assemblages of cyanophages in paddy floodwater of NE China were closer to those in the similar environment of Japanese paddy floodwater than those in Japanese paddy soils, the cyanophage communities in paddy floodwater were still different between the two countries. Chinese paddy floodwater has several phylogenetically novel phage groups, suggesting that paddy field phage communities might differ biogeographically by region/country. Although we tried our best to take samplings at the similar rice growth stage between NE China and Japan, but some factors, such as sampling year, climate condition, and nutrition concentration in paddy floodwaters might result in the formation of different cyanophage communities between the two countries. We acknowledge that the limited sequences observed in this study might not represent most cyanophages in their habits, which need to be further investigated in the future.

The distribution of the *g20* gene of cyanophages varied among different environments, such as lake freshwater, marine water, paddy floodwater, and paddy soil [19], [21], [26], [29]. However, we do not know whether cyanophage community compositions are similar or different among those environments. In this study, the three-dimensional PCoA plot showed that *g20* assemblages from 10 marine waters were located relatively close to each other, but far away from three points of paddy fields and five points of freshwaters. This finding was consistent with the result of T4-type phages [35], suggesting that both T4-type phage and cyanophage community compositions vary among lake freshwater, marine water, and paddy field and that phage community compositions resemble each other in similar environments [35].

It should be noted that the Fig. 4 was constructed by the results of PCR amplification with two primer sets, CPS1/CPS8 and CPS1.1/CPS8.1. Primers CPS1.1/CPS8.1 can amplify the broader range of isolated cyanophages than primers CPS1/CPS8 [37], and the difference in primer specificities between two primer sets resulted in the different cyanophage communities in Lake Bourget conducted by Dorigo et al. [23] and Zhong & Jacquet [45]. However, the two studies were conducted in different years with different sampling strategy and time, therefore, beside of primer, other environmental factors might also cause the differences of cyanophage communities in Lake Bourget between two studies [45]. In addition, although the cyanophage community in Lake Annecy and Bourget estimated using primers CPS1.1/CPS8.1 and other samples estimated using primers CPS1/CPS8, the point of Lake Annecy and Bourget was still located closely to other four points of fresh lakes and far away from points of paddy fields and marine waters, which inferred that the results generated from the two primer sets were comparable.

## Conclusion

In conclusion, a cyanophage capsid assembly protein gene (*g20*) in the paddy floodwater of NE China was successfully amplified with the primers CPS1/CPS8. In total, 54 clones with different *g20* nucleotide sequences were obtained from five paddy floodwaters. The distribution of *g20* sequences in paddy floodwater in NE China was different from that in Japanese paddy fields and was phylogenetically grouped into Clusters α, β, γ and ε. Within Clusters α and β, three new small clusters (PFW-VII∼PFW-IX) were identified in this study. UniFrac analysis of *g20* clone assemblages demonstrated that cyanophage community compositions in paddy floodwater in NE China differed from those in paddy floodwater and soil in Japan. Global analysis of *g20* clone assemblages indicated that the cyanophage community composition varied among marine, lake, paddy field environments.

## Supporting Information

Table S1 **Description of samples sites and the number of** ***g20*** **clones in this study and the corresponding information from original papers used for UniFrac analysis.**
(DOCX)

Table S2 ***P*** **-value test for comparing each point in paddy floodwater in NE China to each point for paddy floodwater and soil in Japan based on UniFrac analysis.**
(DOCX)

Table S3 ***P*** **-value test comparing each point in paddy floodwater to each point for other environments based on UniFrac analysis.**
(DOCX)
