# Supplementary material for: Phylogenetic Distribution of the Capsid Assembly Protein Gene (g20) of Cyanophages in Paddy Floodwaters in Northeast China
Source: PLoS One. 2014 Feb 12;9(2):e88634. doi: 10.1371/journal.pone.0088634 (PMC3922986; doi:10.1371/journal.pone.0088634)
Supplement: Table S1 — Description of samples sites and the number of g20 clones in this study and the corresponding information from original papers used for UniFrac analysis. (DOCX) [file pone.0088634.s001.docx]

**Table S1** Description of samples sites and the number of *g20* clones in this study and the corresponding information from original papers used for UniFrac analysis

| Sample name | Number of clone | Accession numbers | Site description | Reference |
| --- | --- | --- | --- | --- |
| **Marine** |  |  |  |  |
| Polar seas | 16 | AY705095-AY705100, AY7050103,-10,-20,-22,-24,-25,-27,-28,-35,-43 | Water samples from Beaufort Sea (72°30.0'N, 151°20.0'W, 70°12.0'N, 137°0.0'W) and Chuckchi Sea (73°30.0'N 157°00.0'W) in September of 2002 | Short and Suttle 2005 |
| Pacific Ocean | 9 | AY705101,02,-07,-09,-11,-12,-13,-19,-21 | Water sample from coast of Colombia (3°00.60'N, 82°37.90'W), Chile (29°00'S, 77°00'W), Salmon Inlet (49°36.25'N, 123°48.18'W), Pendrell Sound (50°16.24'N, 124°42.80'W) and Malaspina Inlet (50°04.77'N, 124°42.85'W) NE Pacific Ocean  Collect in September of 2002 | Short and Suttle 2005 |
| Atlantic Ocean | 587† | FJ788950-FJ789566 | Water sample from north temperate ocean (47°92.34'N, 14° 61.00'W) to southern boundary of the south Atlantic (28°58.02'S, 06°59.85'W)in September to October of 2004 | Jameson et al. 2011 |
| Gulf Stream | 36 | AY027938-AY027973 | Water sample from Gulf Stream along the edge of the Sargasso Sea (36°24'-37°19'N, 71°20'-37°17'W)  Collected in May of 2000 | Zhong et al. 2002 |
| Sargasso Sea | 70 | AY027974-AY027977; AY28014-AY280078 | Water sample from Sargasso Sea (28°53'-36°47N, 65°04'-71°03'W) in June of 2000 | Zhong et al. 2002 |
| Skidaway | 29 | AY027985-AY028013 | Water sample from Savannah, USA (31°59'N, 81°01'W) in August of 1999 | Zhong et al. 2002 |
| Rhode Island | 25* | AY259244-AY259283 | Water samplecollected from Mount Hope Bay (41°39'N, 71°15'W) from August 1999 through March 2001 | Marston et al. 2003 |
| Chesapeake Bay | 15 | AY152732-AY152746 | Water sample collected from Cape Henlopen (37°07'-39°08' N, 76°07'-77°00' W) from March 2001 to May 2002 | Wang and Chen 2004 |
| Kuwait Coast | 34***** | JX874919-JX874979 | Collected in 2012 | Unpublished, 2013 |
| Shantou Coast in China | 16 | HG326432, HG326441-HG326449  HF565168-HF565173 | Collected in the coastal sea of Shantou, China | Unpublished, 2013  Submitted in June 16,2013 |
| **Freshwater Lake** | | | | |
| Lake Erie | 44 | DQ318388-DQ318399;DQ318401-DQ318432 | Water sample collected in July of 2000, 2002, 2003 | Wilhelm et al. 2006 |
| Lake Cutlus | 9 | AY705108,-29,-31,-33,-34,-36,-37,-41,-44 | Water sample from Lake Cutlus (49°03.45'N, 121°59.05'W) collected in September of 2002 | Short and Suttle, 2005 |
| Lake Bourget | 46 | AY426128-AY426174 | Water sample from Lake Bourget collected in September 2002 to January 2003 | Dorigo et al. 2004 |
| Kranji Reservoir Singapore | 84***** | KC485882-KC485966 | Submitted in Jan 15, 2013 | Unpublished, 2013 |
| Lake Annecy and Bourget | 110 | KC626330- KC626439 | Collected in January 2011 to November 2011 | Zhong, et al. 2013 |
| **Paddy fields** |  |  |  |  |
| JP-PFW | 77 | AB471562-AB471638 | Paddy floodwater sample from Anjo, Japan (34°48'N, 137°30' E) | Wang et al. 2010 |
| JP-PFS | 68‡ | AB560146-AB560215 | Paddy soil sample from Kuroishi, Japan (40°38'N, 140° 35' E), Omagari, Japan(39°29'N, 140°29'E), Anjo, Japan (34°48'N, 137°30' E) | Wang et al. 2011 |
| CN-PFW | 54 | KF017951-KF018004 | Paddy floodwater sample from Da-An (45°36' N, 123°50' E), A-Cheng(45°28' N, 126°58' E), Sui-Hua (46°43' N, E 126°59' E), Lin-Dian (47°18' N, 124°37' E), Jian-San-Jiang (47°14' N, 132°33' E) Northeast China collected in July of 2011 | This study |

***** indicates the number of cyanophage clones obtained with CPS1/CPS8 or CPS1.1/CPS8.1 primer set; † indicates the number of cyanophage clones excluded nonfunctional viral capsid assembly protein (*g20*) gene; ‡ indicates the number of cyanophage clones excluded outgrouped clones
