# Supplementary material for: Phylogenetic Distribution of the Capsid Assembly Protein Gene (g20) of Cyanophages in Paddy Floodwaters in Northeast China
Source: PLoS One. 2014 Feb 12;9(2):e88634. doi: 10.1371/journal.pone.0088634 (PMC3922986; doi:10.1371/journal.pone.0088634)
Supplement: Table S2 — P -value test for comparing each point in paddy floodwater in NE China to each point for paddy floodwater and soil in Japan based on UniFrac analysis. (DOCX) [file pone.0088634.s002.docx]

**Table S2** *P*-value test for comparing each point in paddy floodwater in NE China to each point for paddy floodwater and soil in Japan based on UniFrac analysis

|  | CN-PFW-AC | JP-AnCf-Apr11 | JP-AnCf-Sep04 | CN-PFW-DA | JP-KuCf-Apr13 | JP-KuCf-Jul26 | JP-OmCf-Apr14 | JP-OmCf-Jul27 | JP-PFW-CF | JP-PFW-CF-N | JP-PFW-CM | JP-PFW-NoF | CN-PFW-JSJ | CN-PFW-LD | CN-PFW-SH |
| --- | --- | --- | --- | --- | --- | --- | --- | --- | --- | --- | --- | --- | --- | --- | --- |
| CN-PFW-AC |  | <0.01 | <0.01 | <0.01 | <0.01 | <0.01 | <0.01 | <0.01 | <0.01 | <0.01 | <0.01 | <0.01 | <0.01 | 0.36 | <0.01 |
| JP-AnCf-Apr11 |  |  | 0.01 | <0.01 | <0.01 | <0.01 | <0.01 | <0.01 | <0.01 | <0.01 | <0.01 | <0.01 | <0.01 | <0.01 | <0.01 |
| JP-AnCf-Sep04 |  |  |  | 0.01 | <0.01 | <0.01 | <0.01 | <0.01 | <0.01 | <0.01 | <0.01 | <0.01 | 0.01 | 0.01 | <0.01 |
| CN-PFW-DA |  |  |  |  | <0.01 | 0.01 | <0.01 | <0.01 | 0.02 | <0.01 | <0.01 | <0.01 | 0.01 | <0.01 | <0.01 |
| JP-KuCf-Apr13 |  |  |  |  |  | 0.02 | <0.01 | <0.01 | <0.01 | <0.01 | <0.01 | <0.01 | <0.01 | <0.01 | <0.01 |
| JP-KuCf-Jul26 |  |  |  |  |  |  | 0.02 | 0.01 | 0.01 | 0.03 | <0.01 | 0.01 | 0.01 | 0.01 | <0.01 |
| JP-OmCf-Apr14 |  |  |  |  |  |  |  | 0.77 | <0.01 | <0.01 | <0.01 | <0.01 | 0.01 | 0.01 | <0.01 |
| JP-OmCf-Jul27 |  |  |  |  |  |  |  |  | <0.01 | <0.01 | <0.01 | <0.01 | <0.01 | <0.01 | <0.01 |
| JP-PFW-CF |  |  |  |  |  |  |  |  |  | 0.01 | <0.01 | 0.07 | 0.01 | 0.01 | <0.01 |
| JP-PFW-CF-N |  |  |  |  |  |  |  |  |  |  | 0.04 | 0.05 | <0.010 | <0.01 | <0.01 |
| JP-PFW-CM |  |  |  |  |  |  |  |  |  |  |  | 0.03 | <0.01 | <0.01 | <0.01 |
| JP-PFW-NoF |  |  |  |  |  |  |  |  |  |  |  |  | 0.02 | <0.01 | <0.01. |
| CN-PFW-JSJ |  |  |  |  |  |  |  |  |  |  |  |  |  | <0.01 | <0.01 |
| CN-PFW-LD |  |  |  |  |  |  |  |  |  |  |  |  |  |  | <0.01 |
| CN-PFW-SH |  |  |  |  |  |  |  |  |  |  |  |  |  |  |  |
