# Supplementary material for: Phylogenetic Distribution of the Capsid Assembly Protein Gene (g20) of Cyanophages in Paddy Floodwaters in Northeast China
Source: PLoS One. 2014 Feb 12;9(2):e88634. doi: 10.1371/journal.pone.0088634 (PMC3922986; doi:10.1371/journal.pone.0088634)
Supplement: Table S3 — P -value test comparing each point in paddy floodwater to each point for other environments based on UniFrac analysis. (DOCX) [file pone.0088634.s003.docx]

**Table S3** *P*-value test comparing each point in paddy floodwater to each point for other environments based on UniFrac analysis

|  | Atlantic Ocean | Chesapeake Bay | Gulf Stream | Kranji Reservoir Singapore | Kuwait Coast | Lake Annecy and Bourget | Lake Bourget | Lake Cultus | Lake Laurentian | PFS-JP | PFW-CN | PFW-JP | Pacific Ocean | Polar Seas | Rhode Island | Sargasso Sea | Shantou Coast-CN | Skidaway |
| --- | --- | --- | --- | --- | --- | --- | --- | --- | --- | --- | --- | --- | --- | --- | --- | --- | --- | --- |
| Atlantic Ocean |  | <0.01 | <0.01 | <0.01 | <0.01 | <0.01 | <0.01 | <0.01 | <0.01 | <0.01 | <0.01 | <0.01 | 1.00 | <0.01 | <0.01 | <0.01 | <0.01 | <0.01 |
| Chesapeake Bay |  |  | <0.01 | <0.01 | <0.01 | <0.01 | <0.01 | <0.01 | <0.01 | <0.01 | <0.01 | <0.01 | 0.03 | <0.01 | <0.01 | <0.01 | <0.01 | <0.01 |
| Gulf Stream |  |  |  | <0.01 | <0.01 | <0.01 | <0.01 | <0.01 | <0.01 | <0.01 | <0.01 | <0.01 | <0.01 | <0.01 | <0.01 | 0.74 | <0.01 | <0.01 |
| Kranji Reservoir Singapore |  |  |  |  | <0.01 | <0.01 | <0.01 | <0.01 | <0.01 | <0.01 | <0.01 | <0.01 | <0.01 | <0.01 | <0.01 | <0.01 | <0.01 | <0.01 |
| Kuwait Coast |  |  |  |  |  | <0.01 | <0.01 | <0.01 | <0.01 | <0.01 | <0.01 | <0.01 | 0.08 | <0.01 | <0.01 | <0.01 | <0.01 | <0.01 |
| Lake Annecy and Bourget |  |  |  |  |  |  | <0.01 | <0.01 | <0.01 | <0.01 | <0.01 | <0.01 | <0.01 | <0.01 | <0.01 | <0.01 | <0.01 | <0.01 |
| Lake Bourget |  |  |  |  |  |  |  | <0.01 | <0.01 | <0.01 | <0.01 | <0.01 | <0.01 | <0.01 | <0.01 | <0.01 | <0.01 | <0.01 |
| Lake Cultus |  |  |  |  |  |  |  |  | <0.01 | <0.01 | <0.01 | <0.01 | <0.01 | <0.01 | <0.01 | <0.01 | <0.01 | <0.01 |
| Lak Laurentian |  |  |  |  |  |  |  |  |  | <0.01 | <0.01 | <0.01 | <0.01 | 0.01 | <0.01 | <0.01 | <0.01 | <0.01 |
| PFS-JP |  |  |  |  |  |  |  |  |  |  | <0.01 | <0.01 | <0.01 | <0.01 | <0.01 | <0.01 | <0.01 | <0.01 |
| PFW-CN |  |  |  |  |  |  |  |  |  |  |  | <0.01 | <0.01 | <0.01 | <0.01 | <0.01 | <0.01 | <0.01 |
| PFW-JP |  |  |  |  |  |  |  |  |  |  |  |  | <0.01 | <0.01 | <0.01 | <0.01 | <0.01 | <0.01 |
| Pacific Ocean |  |  |  |  |  |  |  |  |  |  |  |  |  | 0.04 | 1.00 | 1.00 | 0.12 | <0.01 |
| Polar Seas |  |  |  |  |  |  |  |  |  |  |  |  |  |  | <0.01 | <0.01 | <0.01 | <0.01 |
| Rhode Island |  |  |  |  |  |  |  |  |  |  |  |  |  |  |  | <0.01 | <0.01 | <0.01 |
| Sargasso Sea |  |  |  |  |  |  |  |  |  |  |  |  |  |  |  |  | <0.01 | <0.01 |
| Shantou Coast-CN |  |  |  |  |  |  |  |  |  |  |  |  |  |  |  |  |  | <0.01 |
| Skidaway |  |  |  |  |  |  |  |  |  |  |  |  |  |  |  |  |  |  |
